# Expression of Inflammatory Markers RANK, MMP-9 and PTHrP in Chronic Apical Periodontitis from People Living with HIV Undergoing Antiretroviral Therapy

**DOI:** 10.3390/jcm9113611

**Published:** 2020-11-09

**Authors:** Marcio Francisco Pereira, Fabio Ramoa Pires, Luciana Armada, Dennis Carvalho Ferreira, Florence Carrouel, Denis Bourgeois, Lucio Souza Gonçalves

**Affiliations:** 1 Postgraduation Program in Dentistry, Estácio de Sá University, Rio de Janeiro 22790-710, Brazil; dentistamarcio@hotmail.com (M.F.P.); ramoafop@yahoo.com (F.R.P.); luadias@hotmail.com (L.A.); denniscf@gmail.com (D.C.F.); 2 Laboratory “Health Systemic Process”, University Lyon 1, EA4129, 69008 Lyon, France; florence.carrouel@univ-lyon1.fr (F.C.); denis.bourgeois@univ-lyon1.fr (D.B.)

**Keywords:** HIV infections, HAART, periapical periodontitis, receptor activator of NF kappa B, matrix metalloproteinase 9, parathyroid hormone-related protein

## Abstract

To compare the expression of the receptor activator of nuclear factor-kappa B (RANK), matrix metalloproteinase 9 (MMP-9) and parathyroid hormone-related protein (PTHrP) in primary chronic apical periodontitis lesions (CAPLs) between people living with HIV (PLWHIV) undergoing antiretroviral therapy (ART) and HIV- individuals, 32 CAPLs (16 lesions from each group) were submitted to histopathological and immunohistochemical analyses and compared between groups. The majority of the PLWHIV group had undetectable plasma viral loads (*n* = 13; 81.3%). The means of TCD4+ lymphocytes, exposure to HIV-1 and the time of the use of ART were 542.1 cells/mm^3^ (SD = 256.4), 6.3 years (SD = 2.9) and 5.0 years (SD = 2.5), respectively. Of all variables studied, only histopathological diagnosis showed a significant difference between groups (LWHIV: granuloma *n* = 11 (68.0%); cyst *n* = 5 (31.2%); HIV-: granuloma *n* = 15 (93.8%); cyst *n* = 1 (6.2%); *p* = 0.015). When comparing the expressions of the three inflammatory markers between the groups, no significant differences were seen. There was no difference in the expression of RANK, PTHrP and MMP-9 in primary chronic apical periodontitis lesions between PLWHIV under ART and HIV- individuals.

## 1. Introduction

Oral lesions are an especially important marker of immunosuppression [1]. For HIV/AIDS patients, as the CD4+ cell count decreases, the severity and appearance of specific oral lesions are increased [2]. Thus, the spread of HIV/AIDS infection is associated with a high frequency of oral lesions, including hairy leukoplakia, candidiasis, periodontal diseases and Kaposi’s sarcoma [3]. Forty-two HIV-related oral lesions have been identified by the WHO. Classified into three categories, they are categorized as strongly associated lesions, less commonly associated lesions and lesions found during HIV-1 infection [4]. Although more than 20 years have passed, this classification remains valid [1].

Classified in category 3, chronic apical periodontitis lesions (CAPLs) are defined as a persistent inflammation of tissues adjacent to the apex of the tooth root. Pak et al. (2012) estimated at one periapical radiotransparency per patient in industrialized countries which represents a potential source of infection in the order of one billion teeth [5]. The formation of periapical lesions is an immunological response to endodontic infection [6]. Infection is controlled by an adaptive immune response, and an unbalanced immune response is described as an important determinant in the disease outcome [7]. Histologically, a CAPL is mainly characterized as a periapical bone resorption zone, functioning as a cell and cytokine reservoir involved in the control of endodontic infection, as a result of the action of inflammatory mediators that will activate this resorption [8]. In people living with HIV (PLWHIV), there is a reduced ability to respond immunologically to microbial aggression [9], and thus, the evolution of an endodontic infection may develop in a different manner.

With the introduction of antiretroviral therapy (ART), infection with HIV-1 has become a chronic condition [1]. Advances in ART regimens as well as new guidelines and the earlier initiation of treatment have had a substantial impact, manifested by a reduction in secondary infections and diseases, improved overall health and a better quality of life [10]. Thus, the hypothesis that PLWHIV may impact the severity and incidence of oral lesions, since the majority of known PLWHIV are undergoing ART, needs to be re-evaluated. The current question is whether a patient on ART compared to an HIV-negative subject presents different clinical or histological signs of CAPL.

Therefore, a little information about the relationship between CAPLs and HIV-1/ART is available. The present study aimed to compare the expression of the three inflammatory markers, receptor activator of nuclear factor-kappa B (RANK), matrix metalloproteinase 9 (MMP-9) and protein related to the parathyroid hormone PTHrP, in periradicular lesions between PLWHIV undergoing ART and HIV-negative adults.

## 2. Materials and Methods

### 2.1. Case Selection

The study comprised 32 patients (15 males, 17 females) admitted to the Faculty of Dentistry at the Estácio de Sá University, Brazil, between March 2019 and March 2020. Patients were >20 years old and presented at least one CAPL in a tooth indicated for extraction (loss of coronal structure by trauma or extensive dental caries, which made it impossible to restore the tooth), with a clinical and radiographic confirmation. Teeth associated with advanced periodontal lesions or extensive crown destruction allowing permanent contamination from the oral cavity were excluded from the analysis.

Patients with extremely poor oral hygiene, patients with diabetes mellitus, other immunosuppressive diseases (except HIV infection) or severe general health status, patients who received antibiotics within 2 months preceding the extraction of the tooth and users of painkillers, anti-inflammatories or antivirals (except drugs that make up HIV antiretroviral therapy) were not included in the study. The CAPLs that were excluded from this study were due to insufficient size. All PLWHIV were undergoing ART for at least 18 months.

This study protocol was approved by the Ethical Committee for human research of the Estácio de Sá University (CAAE: 26961014.0.0000.5284.), and all patients were informed about the aims, risks and benefits of the study and gave their written informed consent.

### 2.2. Sample Collection and Processing

All teeth were extracted using a straight lever and forceps (Quinelato, São Paulo, Brazil) selected according to the dental configuration. Thirty-two chronic apical periodontitis lesions (CAPLs), 16 of PLWHIV undergoing ART (active group) and 16 of HIV-1 negative adult patients (control group) with systemic health, were obtained by curettage, placed in individual containers containing 10% buffered formalin for 48 h and sent to the Oral Pathology Laboratory (Faculty of Dentistry, Estácio de Sá University). All samples were routinely processed for histologic (hematoxylin-eosin staining in 5-mm sections) and immunohistochemical analyses. The final histologic diagnosis (periapical granuloma or periapical cyst) was rendered by 2 experienced observers.

Only one tooth from each patient presenting with periapical lesion visible on intraoral radiographs was included in this study (PAI score ≥3) [11,12].

### 2.3. Chronic Apical Periodontitis

Chronic apical abscess is an inflammatory reaction to pulpal infection and necrosis characterized by gradual onset, little or no discomfort and an intermittent discharge of pus through an associated sinus tract. Radiographically, there are typically signs of osseous destruction such as a radiolucency. The diagnosis of the discrimination of CAPL from no lesion was made with the use of conventional X-ray imagery. The lesions were classified as small if the diameter was <5 mm in at least one of its variables (length and width) and large if it was ≥5 mm [13]. Teeth were divided into two groups based on whether they exhibited necrotic pulps without prior root canal treatment. Lesions that presented a cavity partially or totally delimited by epithelium were classified as cysts.

### 2.4. Clinical Record

The information through anamnesis included demographic characteristics (ethnicity, smoking, age and gender); the other information collected were medical (medical history, diabetes, hypertension, heart disease, HIV-1 positive serology using the ELISA method and confirmed by the Western blot using ART) and dental (dental history, clinical and radiographic examinations). The periapical radiographs made it possible to determine the size of the CAPL. The CAPL diagnoses were made by two evaluators through the histopathological analysis of the slides stained with hematoxylin and eosin.

### 2.5. Histological Process

The entire histological process was carried out by two researchers (F.R.P. and L.A.), who were blinded to the participants’ HIV status. Histological sections were mounted on silanized slides to carry out the immunohistochemistry reactions following the protocol reported in the literature [14,15,16]. The primary antibodies used were MMP-9 (1: 500, rabbit polyclonal, Dako-A0150, DAKO, Carpinteria, CA, USA), PTHrP (1: 500, monoclonal mouse, sc-53936, Santa Cruz Biotechnology, Dallas, TX, USA) and RANK (1: 200, rabbit polyclonal, sc-9072, Santa Cruz Biotechnology, Dallas, TX, USA).

The LSAB 2 System + HRP System (Dako K0690, DAKO North America, Carpinteria, CA, USA) was used as a secondary antibody. At the end of the process, the liquid DAB (Liquid DAB + Substrate Chromogen System-Dako K3468, DAKO North America, Carpinteria, CA, USA) demonstrated the expression of the cytokines under evaluation. 

Negative controls (omission of the primary antibodies) and positive controls were included in all reactions. Positive control was performed according to the manufacturers’ guidelines: for MMP-9, PTHrP and RANK, normal liver tissue, breast carcinoma and sections of central lesions of giant cells were used, respectively. 

The images were analyzed under an optical microscope (Leica DM500, Heerbrugg, Sweden), and each slide was divided into 5 high-magnification fields (40× magnification) to evaluate the epithelium (periradicular cysts) and connective tissues. Each field received a score between 0 and 2 corresponding to the immunoexpression of each antibody. The expression was considered negative/focal when there were no positive cells or less than 5% of the cells were positive (0 points), weak to moderate when 5% to 50% of the cells were positive (1 point) and strong when more than 50% of the cells were considered positive (2 points). Finally, the averages of the immunoexpression classification were obtained: negative to focal (final average ranging from 0 to 0.5), weak to moderate (ranging from 0.6 to 1.2) and strong (ranging from 1.3 to 2) [14,15].

### 2.6. Statistical Analysis

Data analyses were performed using the SPSS program (Statistical Program for Social Sciences, version 2.1, IBM, São Paulo, SP, Brazil). The normality of continuous variables was verified using the Kolmogorov–Smirnov and Shapiro–Wilk tests, as well as graphical analyses. Nominal variables were expressed as absolute frequency and relative frequency (*n* (%)), while continuous variables were expressed as mean (standard deviation). In the comparison between the HIV (+) and HIV (-) groups, the Mann–Whitney *U* test was used for the continuous variables and the Chi-square test or Fisher’s exact test for the nominal variables. As a sample calculation was not performed, the effect size for each variable was calculated. Thus, after applying the statistical test for the comparison between the groups (obtaining the level of significance for each analysis), the effect size for each variable was calculated. The following interpretation criteria were used for the effect size (d): no effect (0 ≤ d < 0.2), small (0.20 ≤ d < 0.50), medium (0.50 ≤ d < 0.80) and large (d ≥ 0.80) [17]. The size of the effect estimated the magnitude of the difference between the groups for the Mann–Whitney test for each inflammatory marker analyzed. The level of statistical significance established was 5% (*p* < 0.05).

## 3. Results

### 3.1. Characteristics of the HIV-1 Infected and Non-Infected Groups

The PLWHIV group (*n* = 16) consisted of individuals with a mean age of 46.9 years (standard deviation = 10.6), and the majority were male (*n* = 9; 56.3%), white (*n* = 9; 56.3%) and non-smokers (*n* = 15; 93.8%). The non-infected group had a mean age of 53.1 years (standard deviation = 11.1), the majority being female (*n* = 10; 62.5%) and white (*n* = 13; 81.2%), and all were non-smokers. There was no significant difference between the two groups for these variables (*p* > 0.05) (Table 1).

The specific characteristics only related to the PLWHIV group were the following: most had an undetectable plasma viral load (*n* = 13; 81.3%) with a mean of 1062.50 copies/mL (SD = 2619.64) and TCD4 + lymphocytes with a mean of 542.1 cells/mm^3^ of blood (SD = 256.4). The means of exposure to HIV and the duration of ART use were 6.3 years (SD = 2.9) and 5.0 years (SD = 2.5), respectively.

### 3.2. Radiographic and Histopathological Analyses

The size of the periradicular lesions from the radiographs used in this study of PLWHIV ranged from 1.0 mm to 8.0 mm in diameter, with an average diameter of 3.9 mm (SD = 2.4). The frequencies (%) of cysts and granulomas observed were 5 cysts (31.2%) and 11 granulomas (68.8%). The lesion size of the non-HIV infected group varied between 2.0 and 7.0 in diameter, with an average diameter of 4.6 (SD = 1.5). Most of the lesions were diagnosed as granuloma (*n* = 15; 93.8%) and only 1 cyst (6.2%). There was a statistically significant difference only for the frequency of the histopathological diagnosis of the lesion (*p* = 0.015; d = 1.114) (Table 2).

### 3.3. Immunohistochemical Analysis

**Figure 1:**
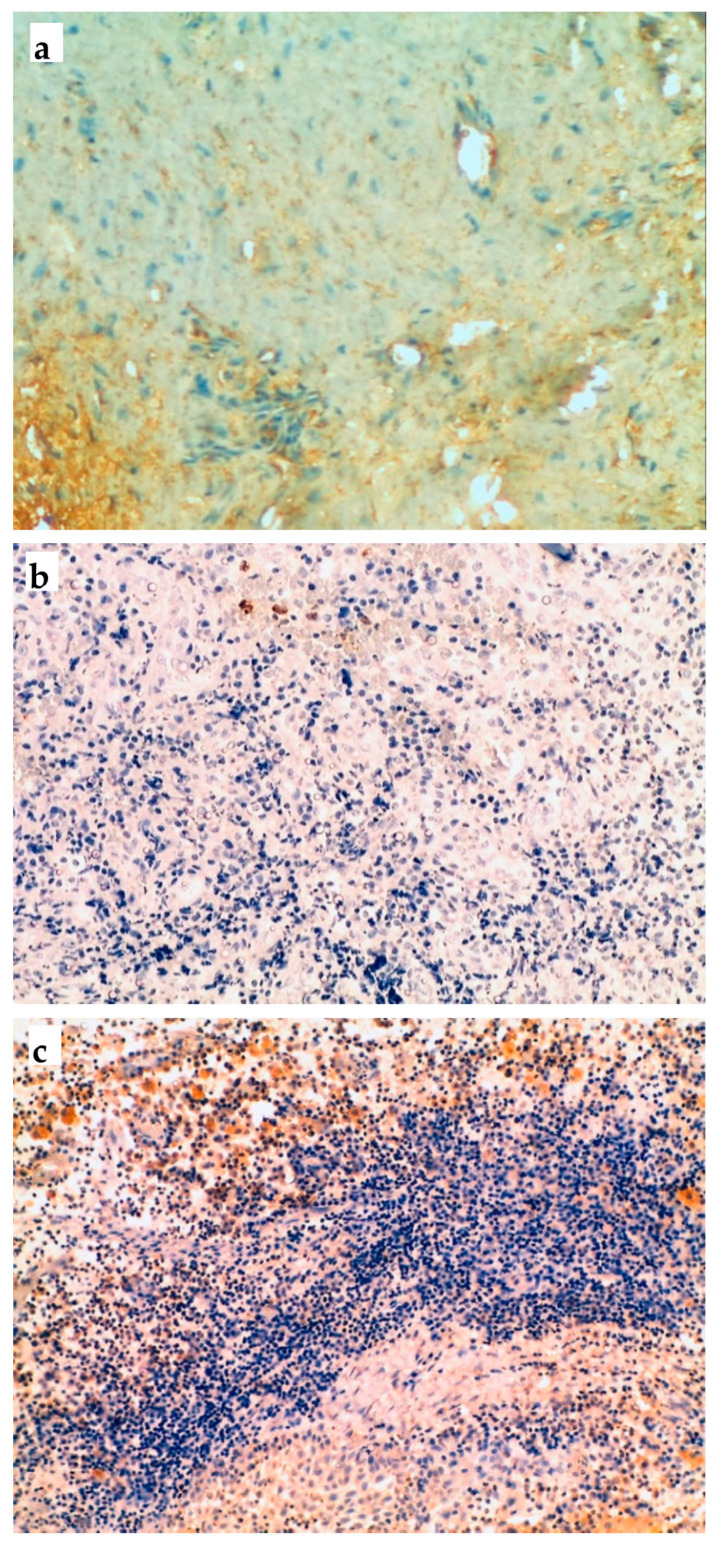
Immunohistochemical expression of cell markers evaluated in the periradicular lesions studied: (**a**) MMP-9 (100× magnification); (**b**) PTHrP (100× magnification); (**c**) RANK (100× magnification).

Immunohistochemical analyses were carried out for each inflammatory marker separately. Table 3 presents the results for the comparison of the MMP-9, PTHrP and RANK expressions between PLWHIV and non-HIV infected patients. The analyses did not show any significant differences between the two groups for the three markers. Figure 1 illustrates the cell labels for MMP9, PTHrP and RANK.

## 4. Discussion

The present study compared the expressions of the inflammatory markers RANK, MMP-9 and PTHrP between PLWHIV and non-HIV infected individuals. The results show no significant differences between the two groups. These findings are probably related to the fact that all PLWHIV were under regular use of ART, which raises the plasma levels of TCD4 + cells and, consequently, regulates the immune system [18], justifying the normality in the expression of the mediators evaluated. 

However, Wattanachanya et al. (2020) demonstrated that PLWHIV, but without the use of ART, have reduced levels of bone formation markers and revealed a direct relationship between the decrease of these markers with the low number of CD4 + cells, thus indicating that bone healing may be altered in PLWHIV [19]. However, it is important to note that the patients evaluated in this latter study did not use ART regularly, which may justify this discrepancy compared to the present study.

According to Desta et al. (2019), one-third of the population living with HIV-1, undergoing ART, had full or partial recovery of the immune system, that is, TCD4 + cell count above 500 cells/mm^3^ of blood, while only 2% of this population remained at critical levels (below 200 cells/mm^3^ of blood) [20]. This shows that the use of ART induces partial or total recovery of the patient’s immune system, which justifies the results of the present study, where the expression of inflammatory mediators was similar to non-infected patients.

The results of the present study agree with Aminoshariae et al. (2017), who found no significant differences in the success rate of endodontic treatment between PLWHIV and non-HIV infected patients [21]. The similarity in the expression of inflammatory mediators between the groups observed in the present study backs up the findings of these authors.

Evaluating patients who were discontinuing ART, Minniear et al. (2014) observed a sharp reduction in the TCD4 + cell count in the first semester after the interruption of this therapy, thus demonstrating the important role played by ART in the patient’s immune system [22]. This important role played by ART is related to the TCD4 + cell count results of the present study, where there was no reduction or difference between PLWHIV and non-HIV infected individuals since all patients with HIV-1 studied here used the therapy regularly. Once again, this reinforces our results that show the similarity of the expression of the inflammatory mediators between the two groups.

Tootla and Owen (2012) reported an increase in the TCD4 + cell count in 87% of the patients evaluated, after starting ART, and did not observe any significant differences in the success rates of endodontic treatment between PLWHIV and non-HIV infected individuals [23]. These results were similar to those found in the studies by Alley et al. (2008) and Quesnell et al. (2005) [24,25]. These latter authors even demonstrated a similarity in the healing of periapical lesions between patients with or without HIV. In addition, Gama et al. (2016) estimated the expression of inflammatory markers in periradicular lesions and found no statistical differences between patients with HIV-1 and those without, similar to the results of the present study [26]. These findings also suggest that patients on the regular use of ART will have the same success rate in endodontic therapy compared to non-infected individuals.

The three mediators studied here were the protein related to the parathyroid hormone (PTHrP), the matrix metalloproteinase 9 (MMP-9) and the receptor activator of nuclear factor-kappa B (RANK). In PLWHIV, there is a reduced ability to respond immunologically to microbial aggression [9], and thus, the evolution of an endodontic infection may develop in a different manner. However, some authors have found no discrepancy in the prognosis of endodontic treatment in these patients [21,27], while others have observed changes in the periapical inflammatory exudate or even in the complexity of the bacterial communities within the root canal [28,29].

PTHrP is a molecule similar to the amino-terminal fraction of parathormone (PTH). According to Wong et al. (2019), PTHrP is an osteolytic and osteoblastic factor, which is widely expressed in bone metastasis of prostate cancer [30]. Its expression is present from birth, showing that the functional formation of the dental arch depends directly on the expression of PTHrP in the role of mediator of the autocrine system responsible for bone modeling and remodeling [31,32].

MMP-9 is an enzyme of the gelatinase class. Its expression is found in areas of active tissue remodeling, and in vascularization, an in vitro study showed its potent angiogenic activity, as well as a noticeable delay in this activity when MMP-9 was not present, thus, MMP-9 can generate angiogenic activators or even inactivate inhibitors of this function [33]. After birth, its expression is present in large cells on bone surfaces, presumably osteoclasts [34]. Recently, MMP-9 expression was related to the number of neutrophils present in the inflammatory zones [35]. MMPs, in general, are involved in apical periodontitis through their relationship with the clinical characteristics of the disease, in the severity of bone destruction, and tend to have an increased expression in lesions greater than 7 mm, especially MMP-1, 2 and 9. In addition, there is a positive correlation between clinical symptoms and an increased expression of MMP-9 [36].

RANK is a cell surface transmembrane receptor, of the TNF family, which is present in osteoclast precursor cells, mature osteoclasts, dendritic cells, fibroblasts and T cells [37]. RANK is activated by its connection with RANKL (receptor activator of nuclear factor-kappa B ligand) which will induce the maturation of pre-osteoclasts, in addition to the activation of mature osteoclasts [38,39]. The expression of RANK is directly related to the development and activation of osteoclasts, in addition to influencing the regulation of the immune system, bone replacement, the development of secondary lymphoid organs and regulation of fever [40]. The expression of this protein above the physiological will lead to exacerbated osteoclastic activity, which will induce deterioration of bone tissue, the same that occurs in some cases of osteoporosis, rheumatoid arthritis and others, including periodontitis [41,42].

The defense of the host against bacterial invasion is based on the immune mechanism, which is responsible for the formation of apical periodontitis [43]. Thus, in patients whose immune system has been damaged, the inflammatory response to endodontic infection and/or the healing of lesions after endodontic treatment may be different [29].

The present study found no statistical difference in the expression of MMP-9 between the groups studied. This finding agrees with the work of Esemu et al. (2019), who evaluated peripheral blood, placental and umbilical cord tissue, without observing a significant difference between PLWHIV and non-HIV patients [44].

Letizia et al. (2019) found changes in the expression of several inflammatory markers in PLWHIV, including the MMP-9 count, when compared to patients without the virus [45]. A similar result was observed by Sufiawati and Tugizov (2018), who demonstrated changes in the MMP-9 count in oral epithelial cells [46]. However, the latter authors conducted an in vitro study, using induced contamination, thus excluding systemic factors that could alter the expression of the inflammatory marker.

The present study did not find any statistical difference in the expression of PTHrP between the groups studied, and this agrees with other authors. According to Bech et al. (2012), the use of ART, associated with the control of calcium and vitamin D, induces a balance of the hormone PTHrP, generating values close to normal, in addition to maintaining the PTHrP expression over time [47]. 

When evaluating the expression of inflammatory markers in periradicular lesions of PLWHIV, the present study demonstrated the ability of the immune system to respond positively to endodontic infection thus making an important contribution to the endodontic literature. However, since PLWHIV without ART were not included in the sample, further studies are necessary to estimate the expression of inflammatory mediators, comparing groups of PLWHIV undergoing ART and PLWHIV groups not using ART. Furthermore, retrospective longitudinal evaluations of these markers should also be performed.

The absence of any statistical differences between the groups indicates similarity in the immune system between patients who have HIV and are undergoing ART and those who do not have HIV. Therefore, from a clinical point of view, these patients will be able to undergo endodontic treatment without fear of a doubtful prognosis due to their systemic condition.

The radicular cyst was significantly more frequent in PLWHIV than non-HIV infected. The development of radicular cysts is associated with the increased expression of inflammatory markers when compared with apical granulomas, activating osteoclasts, and consecutive bone resorption. However, Weber et al. (2019) did not demonstrate a significantly higher expression of RANKL in radicular cysts in comparison with apical granulomas [48]. The three inflammatory markers assessed in the current study (RANK, MMP-9 and PTHrP) are related to bone resorption, but their expressions were not different between PLWHIV and non-HIV infected groups and, therefore, were not associated with the higher frequency of cysts in the first group. New studies need to be developed to clarify this issue.

A limitation of the present study was that it had a small sample, a fact that may have influenced the absence of statistical difference between the groups, with reduced effect size and power of data analysis. The sample size is slightly smaller when compared to previous studies such as Brito et al. (2012) [28], Gama et al. (2016) [26] and De Brito et al. (2015) [29] who evaluated 20 PLWHIV, 17 PLWHIV and 23 PLWHIV, respectively. In addition, as our study evaluated inflammatory markers that have not been previously analyzed in periradicular lesions of PLWHIV, it was not possible to make an appropriate comparative analysis with other studies in the literature.

## 5. Conclusions

The results of the present study demonstrate that there is no difference in the expression of the inflammatory markers RANK (receptor activator of nuclear factor-kappa B), PTHrP (parathyroid hormone-related protein) and MMP-9 (matrix metalloproteinase 9) in primary chronic periradicular lesions between PLWHIV on ART and patients without HIV-1. Management of these lesions will improve the quality of life for HIV/AIDS patients. An awareness of the connection between oral lesions and disease progression is recommended for clinicians treating HIV infection.

## Figures and Tables

**Table 1:** Sociodemographic and behavioral characteristics related to the groups studied. ART: antiretroviral therapy.

Characteristics	HIV+ ART Group (*n* = 16)	HIV- Group (*n* = 16)	*p*	*d* ^1^
**Sociodemographic parameters**				
**Age in years (mean (SD))**	46.9 (10.6)	53.1 (11.1)	0.147	0.558
**Gender-No. (%)**				
Male	9 (56.3)	6 (37.5)	0.719	0.128
Female	7 (43.7)	10 (62.5)		
**Smoker-No. (%)**				
Yes	1 (6.3)	0 (0)	1.00	-
No	15 (93.8)	16 (100)		

^1^ Effect size (d): no effect (0 ≤ d < 0.2), small (0.20 ≤ d < 0.50), medium (0.50 ≤ d < 0.80) and high (d ≥ 0.80).

**Table 2:** Comparison of macroscopic and histopathological characteristics of inflammatory chronic apical periodontitis diagnosed in the two groups studied.

Characteristics	HIV+ ART Group (*n* = 16)	HIV- Group (*n* = 16)	*p*	*d* ^1^
**Lesion size–mean (SD)**	3.9 (2.4)	4.6 (1.5)	0.805	0.087
**Histopathology–**No. (%)				
Granulomas	11 (68.8)	15 (93.8)	0.015	1.114
Cysts	5 (31.2)	1 (6.2)		

^1^ Effect size (d): no effect (0 ≤ d < 0.2), small (0.20 ≤ d < 0.50), medium (0.50 ≤ d < 0.80) and high (d ≥ 0.80).

**Table 3:** Comparison of the expression of the 3 inflammatory markers studied between the PLWHIV and non-HIV infected groups.

Variables	HIV+ ART Group (*n* = 16)	HIV- Group (*n* = 16)	*p*	*d* ^1^
**RANK**	1.2 (0.4); 1.2 (0.6–1.8)	1.1 (0.3); 1.0 (0.6–1.6)	0.396	0.187
**MMP-9**	1.1 (0.5); 1.2 (0.4–1.8)	1.1 (0.5); 0.9 (0.4–1.8)	0.504	0.047
**PTHrP**	0.7 (0.3); 0.8 (0.4–1.2)	0.8 (0.3); 0.8 (0.4–1.2)	0.291	0.140

Data presented as mean (standard deviation) and median (minimum and maximum). RANK: receptor activator of nuclear factor-kappa B; PTHrP: parathyroid hormone-related protein; MMP-9: metallopeptidase-9 matrix. ^1^ Effect size (d): no effect (0 ≤ d < 0.2), small (0.20 ≤ d < 0.50), medium (0.50 ≤ d < 0.80) and high (d ≥ 0.80).
